# Comparative Evaluation of the Marginal Accuracy of Castable Resin Materials Fabricated Using Two Different 3D Printing Methods: An In Vitro Study

**DOI:** 10.7759/cureus.109749

**Published:** 2026-05-27

**Authors:** Marwa Mohd Vazeer, Mahendranadh Reddy, Mahadev Shastry Yelisetty, S Venkat Aditya, Mohd Samad Ali

**Affiliations:** 1 Prosthodontics, Sri Sai College of Dental Surgery, Hyderabad, IND

**Keywords:** 3d printing resin, asiga max, digital light processing (dlp), digital microscope, liquid crystal display (lcd), marginal accuracy, phrozen, standard tesellation language (stl)

## Abstract

Background

Advancements in computer-aided design and computer-aided manufacturing (CAD/CAM) technology have enabled the development of digital workflows for the fabrication of dental restorations. Three-dimensional (3D) printing technologies, such as Liquid Crystal Display (LCD) and Digital Light Processing (DLP), are increasingly being used for the fabrication of castable resin patterns. However, limited information is available regarding their marginal accuracy.

Aim

The aim of this study is to evaluate and compare the marginal discrepancy of castable resin patterns for porcelain-fused-to-metal (PFM) copings fabricated using the LCD (Sonic Mini 4K; Phrozen 3D, Hsinchu City, Taiwan) and DLP (Max 3D; Asiga, Alexandria, New South Wales, Australia) printing methods.

Materials and methods

An in vitro study was conducted using a standardized full crown preparation on a maxillary premolar typhodont tooth. The prepared tooth was scanned, and a coping was digitally designed using CAD software. The design was exported as a standard tessellation language (STL) file for fabrication of castable resin coping patterns using LCD and DLP printers, designated as Group A and Group B, respectively (n = 15 per group). Marginal discrepancy was evaluated at six standardized reference points using a digital microscope. The obtained data were subjected to statistical analysis.

Results

Group B demonstrated lower mean marginal discrepancy values compared with Group A. Statistical analysis revealed a significant difference between the groups (P < 0.001). The mean marginal discrepancy of specimens fabricated using the DLP printer was 48.7 ± 8.6 µm, whereas specimens fabricated using the LCD printer demonstrated a mean marginal discrepancy of 68.5 ± 7.6 µm.

Conclusion

Within the limitations of this in vitro study, castable resin coping patterns fabricated using both LCD and DLP printing methods demonstrated marginal discrepancy values within the selected clinically acceptable range under the conditions of the study. However, the DLP printer exhibited lower marginal discrepancy values compared with the LCD printer.

## Introduction

A precise marginal fit is essential for the biological, mechanical, esthetic, and long-term success of fixed dental restorations [1]. Increased marginal discrepancies may expose the luting cement to the oral environment, resulting in cement dissolution, microleakage, recurrent caries, gingival irritation, and reduced longevity of restorations. Conversely, optimal marginal adaptation improves retention, resistance form, and overall clinical performance of restorations. Although several authors have proposed clinically acceptable marginal discrepancy values ranging from 50 µm to 200 µm, no universally accepted threshold has been established [2].

Traditionally, patterns for metal-ceramic restorations were fabricated using inlay waxes and autopolymerizing resins. However, these materials are associated with dimensional instability and polymerization shrinkage, which may adversely affect marginal accuracy. To overcome these limitations, photopolymerizing materials and digital fabrication techniques have been introduced in restorative dentistry [3].

Three-dimensional (3D) printing, also known as additive manufacturing, is increasingly used in dental laboratory workflows for the fabrication of castable resin patterns. Among vat-polymerization technologies, Liquid Crystal Display (LCD) and Digital Light Processing (DLP) printing methods are commonly employed because of their ability to fabricate complex structures with high precision. In DLP printing, projected light is used to polymerize photosensitive resin through a digital micromirror device, whereas LCD printing uses an LCD screen to selectively transmit light for layer-by-layer polymerization. Variations in printing technology, light source, and resolution may influence the dimensional accuracy and marginal adaptation of fabricated restorations.

Castable resin materials used in these systems are primarily composed of photosensitive acrylate and methacrylate monomers along with photoinitiators and additives. Upon exposure to light of an appropriate wavelength, free-radical polymerization occurs, converting the resin from a viscous liquid into a rigid polymerized structure through the conversion of carbon double bonds (C=C) into covalent carbon-carbon (C-C) bonds [4]. These materials are designed to produce burnout patterns with minimal ash residue after casting. However, polymerization shrinkage during curing may still influence the dimensional accuracy and marginal fit of the fabricated patterns.

Despite the increasing use of LCD and DLP technologies in prosthodontics, limited literature is available comparing the marginal accuracy of castable resin coping patterns fabricated using these two printing systems. Therefore, the present in vitro study aimed to evaluate and compare the marginal discrepancy of castable resin patterns for porcelain-fused-to-metal copings fabricated using LCD and DLP printing methods. The null hypothesis stated that there would be no significant difference in marginal discrepancy between resin coping patterns fabricated using the two printing technologies.

## Materials and methods

Study design

An in vitro comparative study was conducted at Sri Sai College of Dental Surgery, Vikarabad, Telangana, India, from May 24, 2023, to August 20, 2023, to evaluate and compare the marginal discrepancy of castable resin coping patterns fabricated using LCD and DLP 3D printing technologies. A sample size of 15 specimens per group was selected with reference to previously published studies evaluating the marginal discrepancy of resin copings fabricated using digital workflows [4].

Fabrication of the master die

A standardized full veneer crown preparation was performed on a maxillary right first premolar typhodont tooth (Nissin Dental Products Inc., Kameoka City, Kyoto, Japan) for fabrication of porcelain-fused-to-metal coping patterns. The preparation included approximately 1.5-2 mm occlusal reduction, 1-1.5 mm axial reduction, and a shoulder finish line with a bevel of approximately 1-1.2 mm depth. A total occlusal convergence of approximately 6 degrees was maintained throughout the preparation. Standardization of the preparation design was achieved using depth-orientation grooves and digital scanning evaluation.

Six predefined standardized reference points (RPs) representing clinically relevant regions of the tooth preparation were marked below the finish line on the master die to facilitate marginal discrepancy measurements. One RP was positioned at the mid-buccal surface, one at the mid-lingual surface, two on the mesial surface, and two on the distal surface. The facial and lingual RPs were positioned at their respective midpoints, whereas two standardized points were marked on each proximal surface to permit detailed assessment of marginal discrepancy. These markings ensured that all specimens were measured at identical locations, thereby eliminating variability in point selection.

Scanning procedure

**Figure 1:**
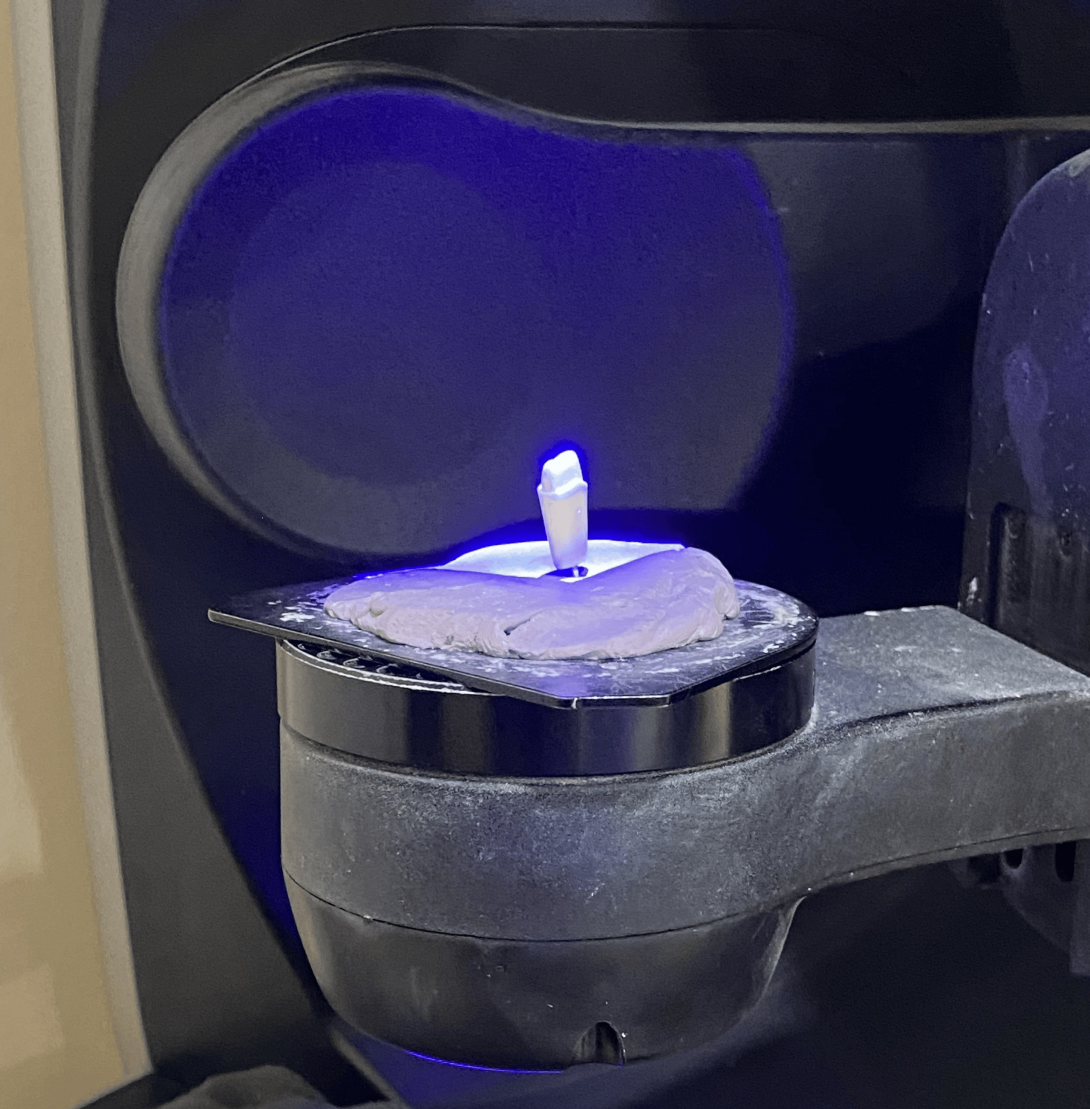
Master die positioned in the laboratory model scanner.

The prepared master die was scanned using the InEos X5 dental laboratory scanner (Dentsply Sirona Inc., Charlotte, North Carolina, United States) after calibration according to the manufacturer’s instructions (Figure 1). A digital scanning protocol was employed to standardize and accurately record all preparation parameters, thereby improving reproducibility. Repeated scans were performed whenever certain surfaces were not clearly captured.

**Figure 2:**
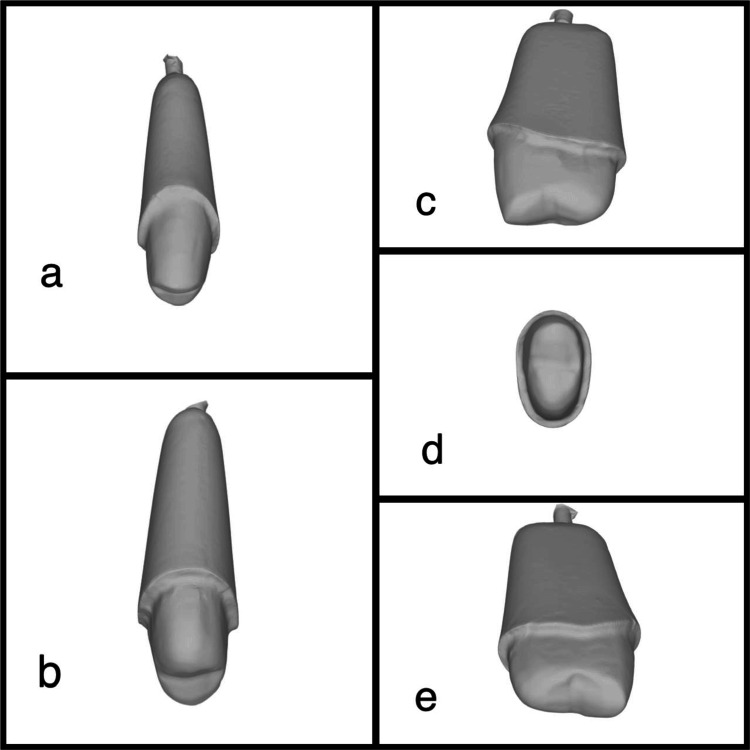
Standard tesellation language (STL) file of master die. (a) buccal view; (b) palatal view; (c) mesial view; (d) occlusal view; (e) distal view

During scanning, anti-glare CAD/CAM scan spray (Telescan Spray; DFS-Diamon GmbH, Riedenburg, Germany) was applied to reduce surface reflectivity and improve scan acquisition accuracy. The scanner utilized five-axis scanning technology, and the finalized scan data were exported as a standard tessellation language (STL) file (Figure 2). The same finalized STL scan data were used for the fabrication of specimens in both Group A and Group B to ensure standardization between the two groups.

CAD designing of copings

The STL file obtained from scanning was imported into Exocad 3.1 Rijeka CAD software (exocad GmbH, Darmstadt, Germany) for designing reduced contour porcelain-fused-to-metal coping patterns with an overall coping thickness of 0.5 mm, with a cement space of 30 µm, and eight supporting structures were added on the occlusal surface anchoring coping to the build platform. The same CAD design was applied to both printers to ensure proper standardization between the two groups.

A total of 30 coping patterns were designed and divided into two groups (n = 15 each): (i) Group A: Copings fabricated using an LCD 3D printer, Sonic Mini 4K (Phrozen 3D, Hsinchu City, Taiwan) and (ii) Group B: Copings fabricated using a DLP 3D printer, Max 3D (Asiga, Alexandria, New South Wales, Australia). Samples were grouped according to the printing technology used.

Printing procedure

Group A (LCD Printing)

The STL files were imported into CHITUBOX V1.8.1 slicer software (CBD-Tech, Shenzhen, China) and printed using the Sonic Mini 4K LCD printer (Phrozen 3D) with 3D Accuprint Model Standard castable resin (D-Tech Limited, Pune, Maharashtra, India). The printer utilized LCD-based vat polymerization with a light wavelength range of 405 nm and XY resolution of 35 µm. The printing parameters were slice thickness and layer height of 0.1 mm, exposure time of 7.5 seconds, lift distance of 5 mm, lift speed of 65 mm/minute, and bottom exposure time of 30 seconds. A total slice count of 106 was obtained, and the total build time was approximately 29 minutes and 34 seconds.

Group B (DLP Printing)

The STL files were imported into Composer 1.3.7 slicer software (Asiga) and printed using the Max DLP printer (Asiga) with 3D Accuprint Model Standard castable resin (D-Tech Limited). The printer utilized DLP-based vat polymerization with a light wavelength range of 385-405 nm. The printing parameters included a slice thickness of 0.10 mm, layer height of 0.15 mm, exposure time of 1.8 seconds, print range height of 9.1 mm, lift speed of 65 mm/minute, and heater temperature of 30°C. A total slice count of 61 was obtained, and the total build time was approximately 7 minutes and 53 seconds.

Post-processing procedure

**Figure 3:**
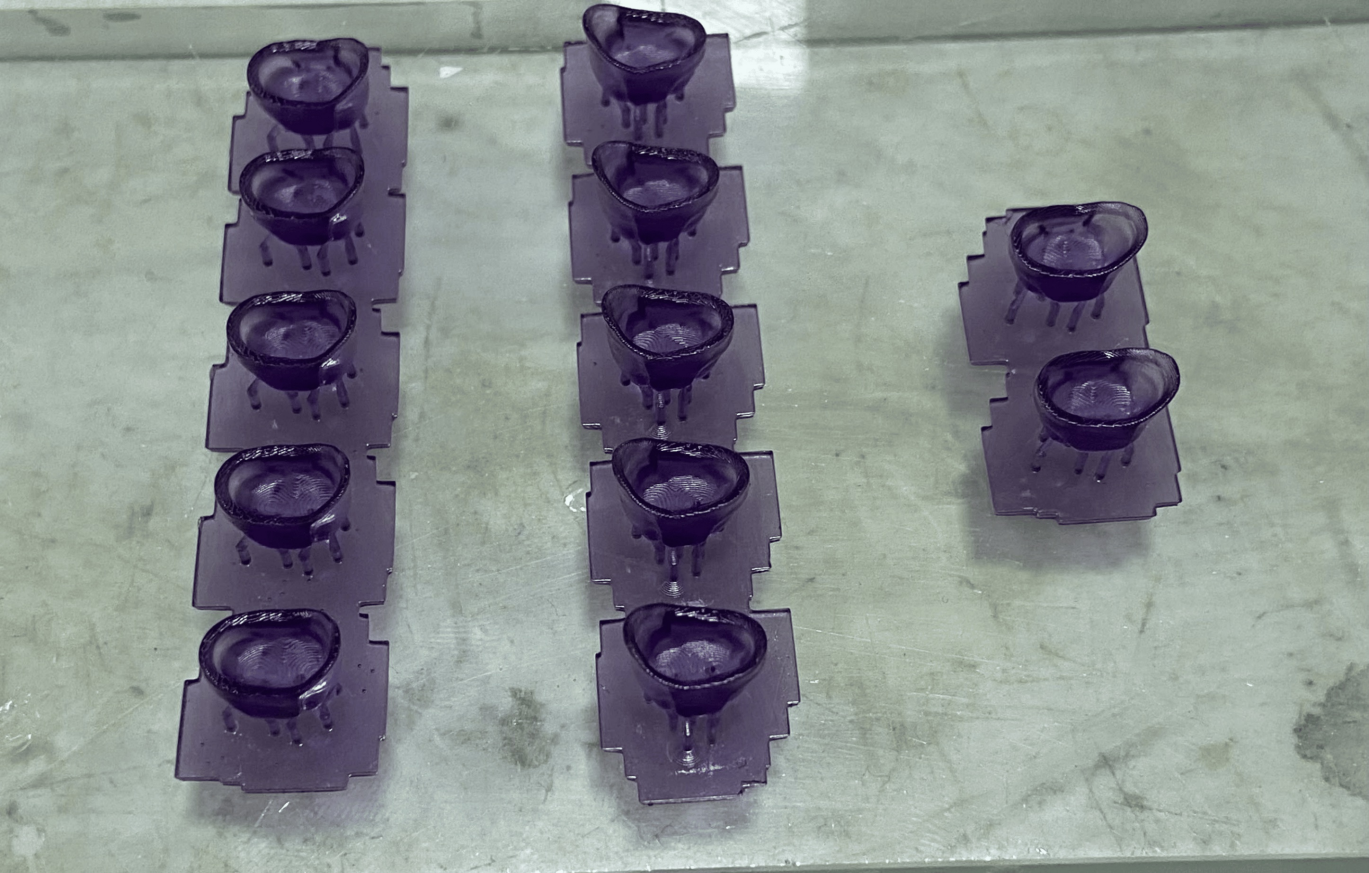
3D-printed resin patterns. 3D: three-dimensional

The printed resin patterns (Figure 3) were cleansed using a magnetic stirrer (Remi 1MLH; Remi Elektrotechnik Ltd., Mumbai, Maharashtra, India) containing isopropyl alcohol (Marble Hill Soaps Ltd, Londonderry, Northern Ireland) at approximately 1420 rpm for two minutes. The specimens were then removed from the build platform and subjected to post-curing in a bre.Lux Power Unit curing device (Bredent Group GmbH & Co.KG, Senden, Germany) with a wavelength range of 370-500 nm for 180 seconds. Supporting structures were subsequently removed manually.

Marginal discrepancy evaluation

The fabricated resin coping patterns were evaluated using an Etzin USB digital microscope (Etzin Private Limited, Mumbai, Maharashtra, India) connected to HiViewSetup1.4 image analysis software (http://www.hvscam.com/soft.asp?lang=en). Calibration was performed using a millimeter calibration ruler under a single standardized system setup prior to the evaluation of all specimens. Measurements were performed at approximately 50× magnification, and all specimens were evaluated within the same standardized calibration session to ensure consistency.

**Figure 4:**
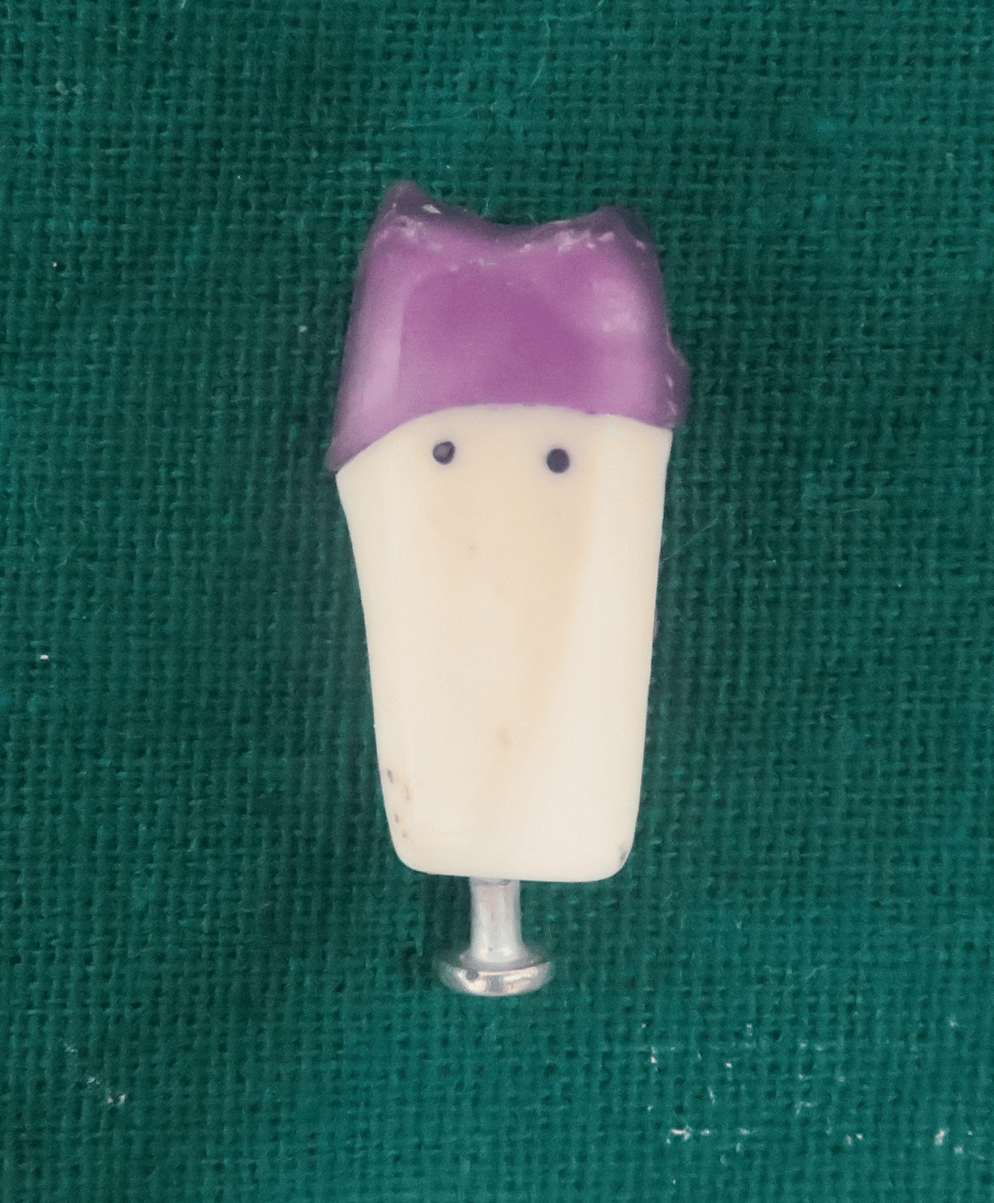
Specimen seated on the master die for microscopic evaluation.

**Figure 5:**
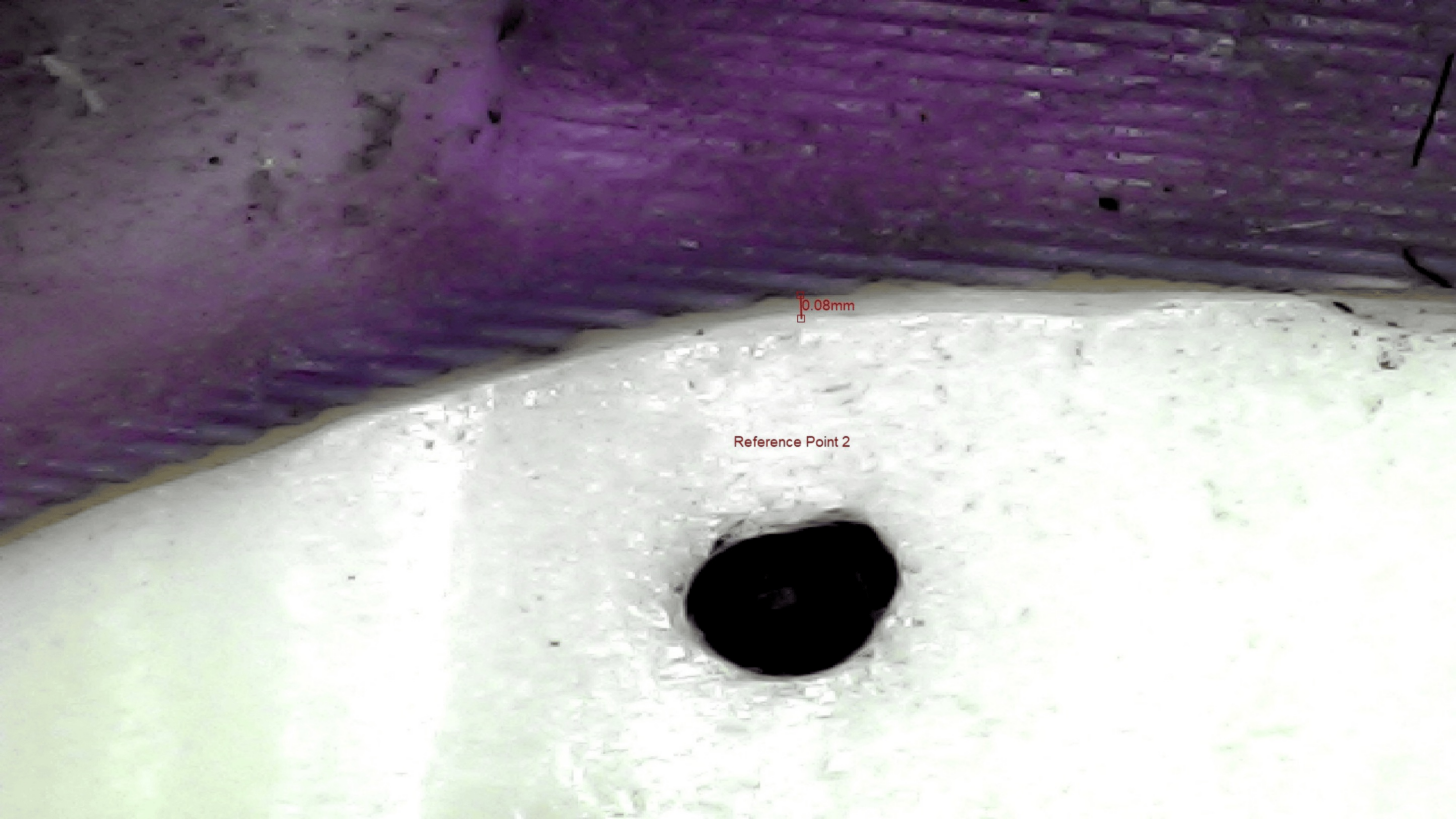
Marginal discrepancy measured using image analyzer software.

Each coping was positioned and seated on the master die (Figure 4), and the vertical marginal discrepancy between the coping margin and finish line was measured at the six predefined RPs (Figure 5). The operator performing the measurements was blinded to the study groups. Measurements at each RP were repeated three times to enhance accuracy, and the mean values were used for statistical analysis.

Statistical analysis

The obtained data were tabulated and subjected to statistical analysis. Normality of data distribution was assessed prior to statistical analysis. An independent samples t-test was used for comparison of normally distributed data between the two groups, whereas the Mann-Whitney U test was applied for non-parametric data. A P value of <0.05 was considered statistically significant.

## Results

The marginal discrepancy of each specimen was evaluated at six standardized RPs using a digital microscope and image analysis software. The obtained measurements were tabulated and statistically analyzed to compare the marginal adaptation of resin copings fabricated using LCD and DLP printers. Table 1 presents the raw marginal discrepancy measurements and the mean marginal discrepancy of Group A specimens fabricated using the LCD printer (Phrozen Sonic Mini 4K). The mean marginal discrepancy values ranged from 55 µm to 77 µm among the specimens.

**Table 1:** Raw marginal discrepancy values and mean marginal discrepancy for Group A samples fabricated using LCD printer (Sonic Mini 4K; Phrozen 3D, Hsinchu City, Taiwan) LCD: Liquid Crystal Display

Group A Sample	RP1 (µm)	RP2 (µm)	RP3 (µm)	RP4 (µm)	RP5 (µm)	RP6 (µm)	Mean (µm)
1	120	90	60	60	30	80	73
2	100	60	60	60	60	70	68
3	90	70	50	40	40	40	55
4	120	80	80	50	50	60	73
5	100	80	40	70	40	70	67
6	90	80	50	80	70	60	72
7	80	60	60	60	50	70	63
8	70	80	90	90	40	80	75
9	90	60	50	80	50	60	65
10	110	60	60	60	90	80	77
11	120	30	50	50	60	60	62
12	130	30	90	40	60	50	67
13	100	70	60	50	40	80	67
14	90	60	90	60	50	60	68
15	100	60	60	50	70	70	68

Table 2 presents the raw marginal discrepancy measurements and the mean marginal discrepancy of Group B specimens fabricated using the DLP printer (Asiga Max 3D). The mean marginal discrepancy values ranged from 35 µm to 62 µm among the specimens.

**Table 2:** Raw marginal discrepancy values and mean marginal discrepancy for Group B samples fabricated using DLP printer (Max 3D; Asiga, Alexandria, Australia) DLP: Digital Light Processing

Group B Sample	RP1 (µm)	RP2 (µm)	RP3 (µm)	RP4 (µm)	RP5 (µm)	RP6 (µm)	Mean (µm)
1	80	50	60	50	20	40	50
2	30	20	70	70	40	30	43
3	80	30	60	70	30	40	52
4	20	10	70	90	30	60	47
5	50	30	60	50	40	70	50
6	60	20	70	50	30	50	47
7	60	10	80	70	30	60	52
8	60	20	80	70	40	60	55
9	80	40	80	80	30	60	62
10	100	20	40	70	40	50	53
11	50	10	50	90	20	20	40
12	110	40	70	40	30	80	62
13	60	10	40	60	30	50	42
14	50	0	50	30	40	40	35
15	30	20	80	50	30	30	40

**Figure 6:**
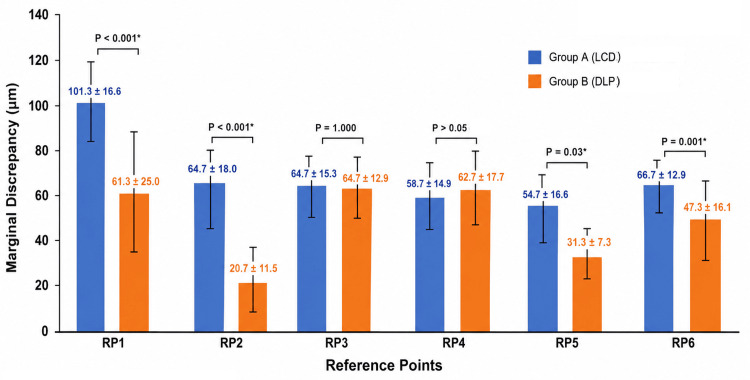
Bar diagram representation showing mean marginal discrepancy of Group A and Group B samples at six reference points *significant (p<0.05; values are given as mean±SD (µm); Independent samples t-test/Mann-Whitney U test RP: reference point

Inter-group comparison of marginal discrepancy at individual RPs is shown in Table 3. Group B demonstrated lower mean marginal discrepancy values than Group A at most RPs. Statistically significant differences were observed at RP1, RP2, RP5, and RP6 (P < 0.05), whereas no statistically significant difference was observed at RP3 and RP4 (Figure 6).

**Table 3:** Intergroup comparison of marginal discrepancy values between Group A (LCD) and Group B (DLP) at different reference points. LCD: Liquid Crystal Display; DLP: Digital Light Processing

Reference Point	LCD (µm), mean±SD	DLP (µm), mean±SD	Test Used	Test Statistic	P value
RP1	101.3 ± 16.6	61.3 ± 25.0	Independent samples t-test	t = 4.63	<0.001
RP2	64.7 ± 18.0	20.7 ± 11.5	Independent samples t-test	t = 6.99	<0.001
RP3	64.7 ± 15.3	64.7 ± 12.9	Independent samples t-test	t = 0.00	1.00
RP4	58.7 ± 14.9	62.7 ± 17.7	Mann–Whitney U test	U = 110.5	>0.05
RP5	54.7 ± 16.6	31.3 ± 7.3	Mann–Whitney U test	U = 67.5	0.03
RP6	66.7 ± 12.9	47.3 ± 16.1	Independent samples t-test	t = 3.56	0.001

The highest mean marginal discrepancy in Group A was observed at RP1 (101.3 ± 16.6 µm), while the lowest mean value was recorded at RP5 (54.7 ± 16.6 µm). In Group B, the highest mean marginal discrepancy was observed at RP1 (61.3 ± 25.0 µm), whereas the lowest mean value was recorded at RP2 (20.7 ± 11.5 µm).

**Figure 7:**
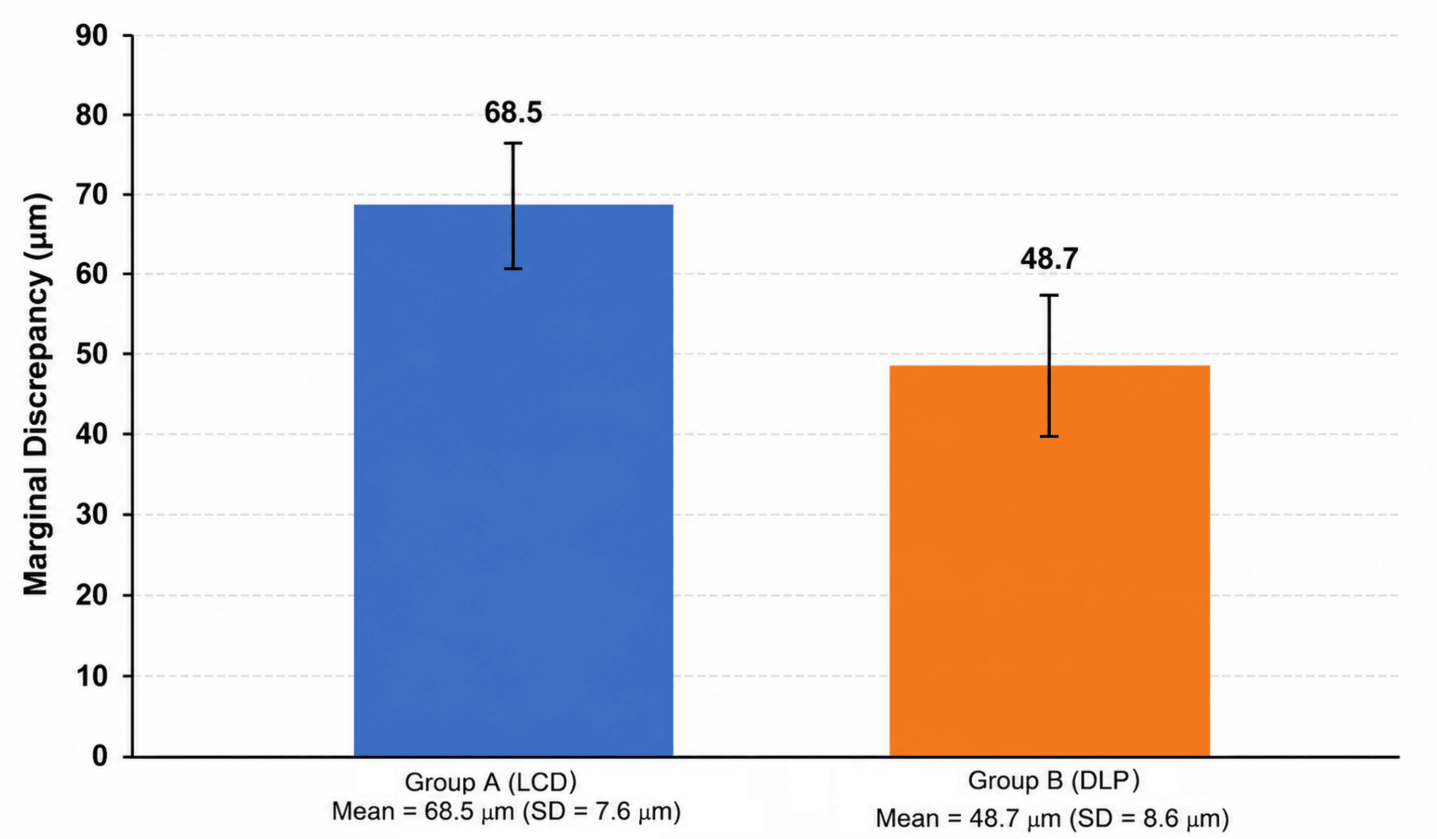
Bar Diagram representation showing overall mean marginal discrepancy of Group A and Group B samples. Independent samples t-test, *t=6.57*; p<0.001 LCD: Liquid Crystal Display; DLP: Digital Light Processing

Table 4 presents the comparison of the overall mean marginal discrepancy between the two groups. The overall mean marginal discrepancy of Group A specimens fabricated using the LCD printer was 68.5 ± 7.6 µm, whereas Group B specimens fabricated using the DLP printer demonstrated a lower overall mean marginal discrepancy of 48.7 ± 8.6 µm (Figure 7).

**Table 4:** Comparison of overall mean marginal discrepancy between Group A and Group B samples. LCD: Liquid Crystal Display; DLP: Digital Light Processing

Group	Mean Marginal Discrepancy (µm)	Standard Deviation (SD)	Test Used	Test Statistic	P value
Group A (LCD)	68.5	7.6	Independent samples t-test	t = 6.57	<0.001
Group B (DLP)	48.7	8.6			

Statistical analysis using an independent samples t-test revealed a highly significant difference between the two groups (P < 0.001), indicating lower marginal discrepancy values in specimens fabricated using the DLP printer under the conditions of the present study.

## Discussion

Metal-ceramic restorations are widely used for full-coverage crowns and fixed partial dentures and remain a standard treatment modality in prosthodontics due to their long-term clinical reliability [5]. The fit of such restorations is determined by both marginal and internal adaptation, with marginal accuracy being critical for clinical success. Marginal discrepancies beyond acceptable limits may contribute to plaque accumulation, gingival inflammation, secondary caries, and eventual restoration failure [6].

The aim of the present study was to compare the marginal discrepancy of PFM coping resin patterns fabricated using castable liquid resin through two vat polymerization 3D printing technologies, namely DLP and LCD.

In this study, D-Tech 3D Accuprint castable resin was used to fabricate resin patterns for PFM copings. According to the manufacturer’s technical datasheet, the material is an ash-free castable resin designed to produce rigid patterns with high dimensional accuracy and minimal deformation during investment and burnout procedures, thereby facilitating accurate casting outcomes under recommended processing conditions. These characteristics are based on manufacturer-reported data and were not independently assessed in the present investigation.

A maxillary right first premolar typhodont tooth was selected as the master die, and tooth preparation was carried out following standard guidelines for PFM crown preparation as described by Shillingburg et al. [1]. Thirty resin copings were fabricated and equally divided into two groups based on the LCD and DLP methods of printing. All specimens were evaluated for marginal discrepancy at six standardized reference points (RPs) to ensure uniform assessment of marginal adaptation. Marginal gaps were measured using a digital microscope and image analysis software. This non-destructive evaluation method enabled direct assessment of vertical marginal misfit without sectioning or cementation, thereby ensuring measurement consistency across all specimens.

The selection of six reference points allowed standardized evaluation of marginal adaptation across all aspects of the coping. Buccal and lingual surfaces are subjected to functional loading during mastication, whereas mesial and distal surfaces contribute to proximal contact and periodontal health maintenance.

In the present study, mean marginal discrepancy values for both groups were within ranges reported in the literature for marginal adaptation of indirect restorations, which vary across studies depending on methodology and evaluation criteria (approximately 25-120 µm) [7-10]. Group A (LCD) exhibited a higher mean marginal discrepancy (68.5 ± 7.6 µm), whereas Group B (DLP) demonstrated a lower mean value (48.7 ± 8.6 µm), indicating improved marginal adaptation in the DLP group. Statistically significant differences between the groups were observed at RP1, RP2, RP5, and RP6, whereas RP3 and RP4 demonstrated no significant inter-group difference.

The null hypothesis stating that no difference exists between DLP and LCD fabricated copings was rejected, as statistical analysis demonstrated a significant difference between the groups (P < 0.001).

The difference in marginal adaptation between the two groups may be associated with the inherent differences in vat polymerization technologies. DLP systems employ a projected light source that cures each layer simultaneously, while LCD systems use masked ultraviolet light passing through an LCD panel. Differences in light distribution characteristics and resolution capacity between these systems can influence polymerization uniformity and dimensional accuracy, which may account for variations in marginal adaptation. These findings are in accordance with the observations reported by Hai et al. [11].

The higher marginal discrepancy observed at RP1 (mid-buccal region) may be associated with procedural factors related to digitization and surface preparation. The application of scan spray, used to improve surface detectability during optical scanning, may introduce minor variability due to its coating characteristics, which is in accordance with the findings reported by Ahmadi et al. [12]. Any non-uniformity in its application could have influenced margin capture under the specific experimental conditions of this study. This observation should therefore be interpreted as a potential source of experimental variability rather than a limitation of the scanning system itself.

Arora et al. [13] reported a mean marginal discrepancy of 101.67 ± 4.88 µm for 3D-printed resin patterns, which falls within the range observed in the present study. Similarly, Arora et al. [14] reported marginal gap values of 82.21 ± 15.26 µm for the 3D-printed resin group, which are comparable with the findings of the current investigation.

Although both groups demonstrated marginal discrepancy values within clinically acceptable ranges previously reported in the literature, it should be noted that reported thresholds for marginal adaptation vary across studies, and no single universally accepted cutoff exists. Therefore, interpretation of the present findings is based on previously published ranges rather than a fixed standard.

Limitations of the study include its in vitro design and the controlled laboratory conditions under which all specimens were fabricated and evaluated. Clinical conditions such as salivary contamination, dynamic functional loading, and intraoral variability were not simulated. Digital scanning procedures may be influenced by system resolution and surface capture characteristics. Variations related to polymerization shrinkage, printing orientation, and post-curing procedures may also influence marginal adaptation. Additionally, the relatively small sample size may limit the generalizability of the findings. Therefore, these factors should be considered as methodological variables inherent to the experimental setup rather than direct limitations of the scanning system. As a result, the findings may not fully replicate clinical conditions and should be interpreted accordingly.

Further investigations involving broader sample sizes and clinical evaluations are warranted to enhance understanding of the influence of vat polymerization technologies on the marginal accuracy of 3D-printed resin patterns.

## Conclusions

Within the limitations of this in vitro study, specimens fabricated using the DLP 3D printer demonstrated significantly lower marginal discrepancy compared to those produced using the LCD printer. The mean marginal discrepancy was lower for the DLP group. However, both groups showed marginal discrepancy values within the predefined acceptable range under the conditions of this study. These findings suggest that vat polymerization printing technology may influence the marginal accuracy of resin copings, with DLP demonstrating comparatively better performance. Nevertheless, as this investigation was limited to in vitro evaluation of resin copings, caution should be exercised while extrapolating the results directly to clinical outcomes. Further long-term in vivo and in vitro studies are required to validate these findings and to assess the clinical relevance and performance of these materials under functional conditions.

## References

[1] Shillingburg HT, Sather DA, Wilson EL, Cain JR, Mitchell DL, Blanco LJ, Kessler JC (2012). Fundamentals of Fixed Prosthodontics. Chicago: Quintessence Publishing Co.

[2] Martínez-Rus F, Suárez MJ, Rivera B, Pradíes G (2011). Evaluation of the absolute marginal discrepancy of zirconia-based ceramic copings. J Prosthet Dent.

[3] Jain R, Supriya Supriya, Bindra S, Gupta K (2016). Recent trends of 3D printing in dentistry - a review. Ann Prosthodont Restor Dent.

[4] Rajagopal P, Chitre V, Aras MA (2012). A comparison of the accuracy of patterns processed from an inlay casting wax, an auto-polymerized resin and a light-cured resin pattern material. Indian J Dent Res.

[5] Pettenò D, Schierano G, Bassi F, Bresciano ME, Carossa S (2000). Comparison of marginal fit of 3 different metal-ceramic systems: an in vitro study. Int J Prosthodont.

[6] Hunter AJ, Hunter AR (1990). Gingival margins for crowns: a review and discussion. Part II: discrepancies and configurations. J Prosthet Dent.

[7] McLean JW, von Fraunhofer JA (1971). The estimation of cement film thickness by an in vivo technique. Br Dent J.

[8] Christensen GJ (1966). Marginal fit of gold inlay castings. J Prosthet Dent.

[9] Palomo F, Peden J (1976). Periodontal considerations of restorative procedures. J Prosthet Dent.

[10] Ostlund LE (1985). Cavity design and mathematics: their effect on gaps at the margins of cast restorations. Oper Dent.

[11] Hai PN, Son TM, Anh NV, Ngoc VT, Tra NT (2023). Effect of horizontal resolution of printer on trueness of 3D-printed provisional crown: an in vitro study. Eur J Gen Dent.

[12] Ahmadi E, Tabatabaei MH, Sadr SM, Atri F (2020). Comparison of the marginal discrepancy of PFM crowns in the CAD/CAM and lost-wax fabrication techniques by triple scanning. Dent Med Probl.

[13] Arora A, Yadav A, Upadhyaya V, Jain P, Verma M (2018). Comparison of marginal and internal adaptation of copings fabricated from three different fabrication techniques: an in vitro study. J Indian Prosthodont Soc.

[14] Arora O, Ahmed N, Maiti S (2022). Comparison of the marginal accuracy of metal copings fabricated by 3D-printed resin and milled polymethyl methacrylate - an in vitro study. J Adv Pharm Technol Res.

